# First-In-Human Study to Assess the Safety, Pharmacokinetics, and Pharmacodynamics of SHR2285, a Small-Molecule Factor XIa Inhibitor in Healthy Subjects

**DOI:** 10.3389/fphar.2022.821363

**Published:** 2022-02-10

**Authors:** Rui Chen, Xiaoduo Guan, Pei Hu, Yanli Dong, Yi Zhu, Tengfei Zhang, Jianjun Zou, Shuyang Zhang

**Affiliations:** ^1^ Clinical Pharmacology Research Center, Peking Union Medical College Hospital, Chinese Academy of Medical Sciences and Peking Union Medical College, Beijing, China; ^2^ Jiangsu Hengrui Pharmaceuticals Co, Ltd, Shanghai, China; ^3^ Department of Cardiology, Peking Union Medical College Hospital, Chinese Academy of Medical Sciences, Beijing, China

**Keywords:** anticoagulant, factor XI inhibitor, first-in-human trial, SHR2285, pharmacokinetics, pharmacodynamics

## Abstract

**Background:** Targeting factor XI (FXI) is a promising therapeutic strategy for the treatment and prevention of thrombosis without increasing the risk of bleeding. Here, we assessed the safety, pharmacokinetics (PK), and pharmacodynamics (PD) of SHR2285, a novel FXIa inhibitor, in healthy subjects.

**Methods:** In this randomized, double-blinded, placebo-controlled, dose-ascending single-dosing trial (NCT03769831), eligible volunteer subjects receive either SHR2285 or placebo in a 3:1 ratio. Subjects assigned to the SHR2285 group received a single oral dose of SHR2285 at 50 mg, which was subsequently escalated to 100 mg, 200 mg, and 400 mg. Safety, pharmacokinetics, and pharmacodynamics parameters were assessed. All subjects were followed for 6 days.

**Results:** SHR2285 was well tolerated. All adverse events were grade 1, and there was no evidence of bleeding events. The PK results revealed a rapid onset of action of SHR2285 (median time to maximum plasma concentration [T_max_] in different dose groups ranged 3.0–4.0 h) and the mean half-life ranged from 7.6 to 15.8 h. The metabolite SHR164471 had a slightly longer T_max_ than the parent SHR2285, reaching a peak at a median of 6.0–7.0 h, and its mean half-life were 10.1–14.7 h in different dose groups. The sums of the area under the concentration–time curve from zero to time infinity of SHR2285 and SHR164471 in the 200 and 400 mg groups were similar, indicating the sum pharmacological activity of SHR2285 and SHR164471 showed a saturation trend between 200 and 400 mg. PD analysis showed that the inhibition of FXI activity was synchronized with prolonged activated partial thromboplastin time after SHR2285 administration, but the serum prothrombin time and international normalized ratio levels were not affected by SHR2285.

**Conclusion:** SHR2285 demonstrated favorable safety, PK, and PD profiles in the dose range of 50 mg–400 mg. This first-in-human study supports the further development of SHR2285 for indications requiring anticoagulation.

**Clinical Trial Registration**: https://clinicaltrials.gov/ct2/show/NCT03769831, identifier [NCT03769831].

## Introduction

Thromboembolic disease has a high incidence rate and carries high mortality and disability rates (Wendelboe and Raskob, 2016). The clinical benefits of anticoagulant therapy in thrombosis treatment and prevention have been clinically validated (Mackman et al., 2020). Traditional anticoagulants including heparins, vitamin K antagonists (e.g., warfarin), direct thrombin inhibitors (e.g., bivalirudin and dabigatran), and direct activated factor X (FXa) inhibitors (e.g., apixaban, edoxaban, and rivaroxaban) have shown encouraging efficacies for reducing thrombosis (Mackman et al., 2020), but excessive bleeding is the most concerning complication of these drugs (Shoeb and Fang, 2013; Inohara et al., 2018). Therefore, reducing the risk-to-benefit ratio is still the key objective in antithrombotic drug development.

Activated coagulation factor XI (FXIa) is emerging as an exciting therapeutic target for anticoagulant therapies (Lowenberg et al., 2010; Schumacher et al., 2010; Gailani and Gruber, 2016; Weitz and Fredenburgh, 2017; Mackman et al., 2020). Factor XI is a key component in maintaining the intrinsic coagulation pathway. Moreover, FXI-induced thrombin can activate FXI in a feedback loop, and activated FXI further promotes thrombin production, which amplifies the coagulation cascade (Gailani and Renne, 2007). In FXI-deficient animal models or patients, there is no evidence of obvious bleeding by targeting FXIa, which supports FXIa as a potential therapeutic target for anticoagulant therapy (Mackman et al., 2020). Several candidate drugs targeting FXIa have entered the early stage of clinical development, including small-molecule FXI inhibitors (JNJ 70033093 [BMS-986177], BAY 2433334, ONO-7684, BMS-962212, and EP-7041) (Hayward et al., 2017; Perera et al., 2018; X.; Wang et al., 2020; Beale et al., 2021; Thomas et al., 2021), FXI antibodies (abelacimab [MAA868], osocimab [BAY1213790], and xisomab [AB203]) (Koch et al., 2019; Lorentz et al., 2019; Thomas et al., 2019; Weitz et al., 2020), and a FXI-antisense oligonucleotide (Buller et al., 2015). However, no FXIa-targeted drugs have been approved for clinical use.

SHR2285 is an oral, novel small molecular compound that selectively inhibits FXIa. The half-life of small-molecule drugs is commonly shorter than that of monoclonal antibodies, and oral administration is convenient. Preclinical studies have shown that it reduced thrombosis and prolonged the activated partial thromboplastin time (APTT) with no bleeding events in the New Zealand rabbit model (data on file, Hengrui). Based on this evidence, we conducted this first-in-human study to evaluate the tolerability, safety, pharmacokinetics (PK), and pharmacodynamics (PD) of SHR2285 in healthy subjects.

## Methods

### Study Population

Eligible subjects were required to be healthy, aged 18–45 years, and have a body mass index (BMI, calculated by kg/m^2^ where kg is a person’s weight in kilograms and m^2^ is the height in meters squared) range of 18–28 kg/m^2^. The key exclusion criteria included known hypersensitivity to other ingredients besides SHR2285 in the SHR2285 tablets; history of known coagulation disorders; use of anticoagulants or antiplatelet drugs within 1 month before enrollment; aspartate aminotransferase, alanine aminotransferase, γ-glutamyl transferase, or total bilirubin levels ≥1.5× upper limit of normal (ULN) at screening; serum creatinine levels above ULN; and a history of smoking within 1 month or ≥15 g alcohol/day within 1 week before study medication administration.

### Study Design and Treatment

This phase 1 study was a randomized, double-blinded, placebo-controlled, dose-ascending, single-dosing trial of SHR2285 in healthy subjects (NCT03769831). SHR2285 was given orally as tablets with 200 ml water to fasted subjects. Seven dose cohorts (50, 100, 200, 400, 600, 800, and 1,000 mg) were preplanned. The maximum recommended starting dose was calculated based on the no observed adverse effect levels and SHR2285 exposure levels in animal studies, according to the guidelines of China National Medical Products Administration for “Estimating the maximum recommended starting dose of drugs in initial clinical trials of healthy adult volunteers.” The starting dose cohort (50 mg, chosen based on preclinical safety data) enrolled four subjects (three received SHR2285 and one received placebo). The first subject was set as an open-blind sentinel and was given 50 mg SHR2285. After justification of safety and tolerability in this subject for ≥48 h, the other three subjects were randomized in a 2:1 ratio to receive either SHR2285 50 mg (n = 2) or placebo (n = 1). Dose escalation was performed only after the third-party investigator confirmed that the termination criteria had not been met according to the 7-days safety and tolerance observation of the preceding dose cohort. Except for the 50-mg group, each of the other dose groups enrolled eight patients: two volunteers were set as sentinel subjects (randomized 1:1 to receive SHR2285 [n = 1] or placebo [n = 1]) and were evaluated for safety for ≥48 h, followed by the further enrollment of other six subjects (randomized 5:1 to receive SHR2285 [n = 5] or placebo [n = 1]). Randomization was performed using SAS v9.4 software (SAS Inc., Cary, NC, USA).

If ≥ 50% of the subjects in any dose group experienced grade 2 toxicity related to the study drug and had relevant clinical manifestations, or ≥33% of the subjects in any dose group had any grade 2 toxicity related to the study drug, the assessment in this dose group and further dose escalation was terminated. Then the investigator would discuss with the sponsor to choose to terminate the trial or return to the mean dose of this dose and the lower dose and keep the number of cases unchanged to continue the trial. If the above termination criteria were met again, the study would be terminated. Other possible reasons for study termination or suspension included major deviation or human error during the test implementation, which seriously affected the test quality and made it difficult to achieve the study purpose; termination requested by the sponsor on the premise of fully protecting the rights and safety of the subjects; termination requested by the ethics committee or investigator to protect the subject; termination requested by the China National Medical Products Administration; or the sponsor and investigator decided not to escalate the dose to a higher dose according to the obtained FXIa activity inhibition data.

### Safety Assessment

Adverse events (AEs) were assessed throughout the 6 days after study drug administration. AEs were classified according to the Medical Dictionary for Regulatory Activities (MedDRA, v21.1) and graded based on National Cancer Institute’s Common Terminology Criteria for Adverse Events (NCI-CTCAE, v5.0). Vital signs and electrocardiograms were assessed at baseline and 4 h, 8 h, 12 h, 24 h, 48 h, and 6 days postdose. Clinical laboratory tests and physical examinations were also performed.

### PK Analysis

Blood samples (4 ml venous blood at each time point) for PK analyses were collected at predose and 0.5 h, 1 h, 1.5 h, 2 h, 3 h, 4 h, 5 h, 6 h, 8 h, 10 h, 12 h, 24 h, and 48 h after SHR2285 administration. Plasma concentrations of SHR2285 and its metabolite SHR164471 were measured using liquid chromatography–tandem mass spectrometry. The calibration ranges of SHR2285 and SHR164471 were 5 ng/ml (lower limit of quantification) to 5,000 ng/ml. Samples >5,000 ng/ml were diluted to concentrations within the calibration range. For SHR2285 and SHR164471, the mean inter-assay accuracies of back-calculated concentrations in calibrators were 95.7%–104.2% and 95.4%–102.5%, respectively, and the corresponding precision were ≤4.6% and ≤5.4%. Quality control samples (15–4,000 ng/ml) were determined with accuracies of 99.9%–100.8% and 101.2%–103.3%, and precision of ≤4.7% and ≤8.3%, respectively, for both analytes.

The evaluated PK parameters included area under the plasma concentration–time profile (AUC), time to maximum plasma concentration (T_max_), maximum plasma concentration (C_max_), half-life (t_1/2_), mean residence time (MRT), apparent total clearance (CL/F), and apparent volume of distribution (V/F). PK analyses were performed in Frontage Laboratories Co. Ltd (Shanghai, China).

### PD Analysis

Blood samples for PD analysis were collected at predose and 1 h, 1.5 h, 2 h, 4 h, 6 h, 8 h, 12 h, 24 h, and 48 h after SHR2285 administration. FXI activity was measured with 5 ml of venous blood at each time point; activated partial thromboplastin time (APTT), prothrombin time (PT), and international normalized ratio (INR) were evaluated with 3 ml of blood at each time point. FXI activity was assessed using an automated coagulometer (CS-1300, Sysmex, Kobe, Japan) at the Institute of Blood Transfusion, Chinese Academy of Medical Sciences of China; APTT, PT, and INR were determined by transmission turbidimetry at the Peking Union Medical College of China.

### Statistical Analysis

No formal statistical calculation was used to predetermine the sample size. Disposition and baseline demographics were analyzed in all enrolled subjects. Safety was analyzed in all subjects who received at least one dose of SHR2285. Plasma drug concentration and PK parameter analysis was performed in subjects who received at least one dose of SHR2285 and had at least one qualified plasma drug concentration and PK parameter result. PD analysis was performed in subjects who received at least one dose of SHR2285 and had non-missing baseline data and at least one qualified PD assessment data point.

Baseline demographics, safety results, PK, and PD were summarized descriptively. Geometric average and geometric coefficient of variation were also provided for PK parameters. Plasma drug concentrations are graphically presented as individual, mean, and median plasma drug concentration–time plots. AUC was calculated using a non-compartment model. Dose-normalized PK parameters on log transformation were analyzed for dose linearity between SHR2285 and its metabolites using analysis of variance (ANOVA), and the box–whisker plots were generated for the correlation analyses of PK parameters and doses. All statistical analyses were performed using SAS v9.4 and Phoenix v8.0 (Certara, Princeton, NJ, USA).

## Results

### Patients

**FIGURE 1:**
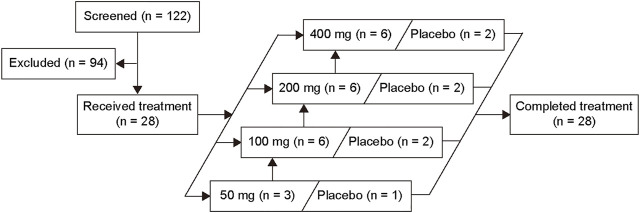
Volunteer disposition.

A total of 28 eligible subjects (50 mg, 4 subjects; 100 mg, 8 subjects; 200 mg, 8 subjects; and 400 mg, 8 subjects) were enrolled in this study, of which 7 subjects received placebo and 21 received the study drug SHR2285. All subjects completed the study (Figure 1). Baseline characteristics are presented in Table 1.

**TABLE 1:** Baseline demographics.

	Placebo (n = 7)	50 mg (n = 3)	100 mg (n = 6)	200 mg (n = 6)	400 mg (n = 6)	All subjects (n = 28)
Age (years)						
Mean (SD)	31.7 (7.2)	35.0 (4.4)	31.3 (10.8)	32.7 (7.9)	25.5 (3.0)	30.9 (7.6)
Median (range)	33.0 (19–40)	33.0 (32–40)	30.5 (21–43)	33.5 (22–40)	26.0 (22–29)	29.0 (19–43)
Gender, n (%)						
Male	6 (85.7)	2 (66.7)	5 (83.3)	3 (50.0)	5 (83.3)	21 (75.0)
Female	1 (14.3)	1 (33.3)	1 (16.7)	3 (50.0)	1 (16.7)	7 (25.0)
Weight (kg)						
Mean (SD)	69.8 (6.3)	66.7 (6.6)	60.5 (8.8)	67.4 (6.7)	60.5 (4.9)	65.0 (7.5)
Median (range)	69.3 (61.6–79.8)	63.4 (62.5–74.3)	56.7 (54.0–77.5)	64.8 (62.6–80.1)	59.8 (54.8–69.7)	63.0 (54.0–80.1)
Height (cm)						
Mean (SD)	170.0 (5.7)	162.0 (10.2)	169.3 (2.9)	167.5 (7.0)	173.2 (4.9)	169.1 (6.3)
Median (range)	172.0 (159–176)	164.0 (151–171)	169.5 (165–174)	163.5 (162–177)	173.5 (165–180)	170.0 (151–180)
BMI (kg/m^2^)						
Mean (SD)	24.2 (1.9)	25.5 (2.3)	21.1 (2.9)	24.0 (1.5)	20.2 (1.6)	22.8 (2.7)
Median (range)	25.1 (20.3–25.8)	25.4 (23.2–27.8)	20.3 (19.1–26.8)	23.9 (21.5–25.7)	19.8 (18.7–23.3)	23.4 (18.7–27.8)
Alcohol use, n (%)						
Never	7 (100.0)	3 (100.0)	6 (100.0)	6 (100.0)	6 (100.0)	28 (100.0)
Smoking history, n (%)						
Never	6 (85.7)	3 (100.0)	5 (83.3)	6 (100.0)	6 (100.0)	26 (92.9)
Former	1 (14.3)	0	1 (16.7)	0	0	2 (7.1)
Current	0	0	0	0	0	0

Data are n (%) unless otherwise specified.

### Safety

Safety was assessed in all 28 subjects enrolled. A total of 15 subjects experienced at least one AE, including 12 (57.1%) subjects in the SHR2285 group and 3 (42.9%) subjects in the placebo group (Table 2). In subjects receiving SHR2285, the most common AEs were increased bilirubin conjugated (19.0%, n = 4) and occult blood positive (19.0%, n = 4). All AEs were grade 1; no grade 2 or greater AEs were reported. All AEs were recovered or resolved without special interventions. No serious AE or deaths occurred.

**TABLE 2:** Adverse events.

	Placebo (n = 7)	50 mg (n = 3)	100 mg (n = 6)	200 mg (n = 6)	400 mg (n = 6)	All SHR2285 (n = 21)
Adverse events of any cause, n (%)	3 (42.9)	1 (33.3)	3 (50.0)	4 (66.7)	4 (66.7)	12 (57.1)
Biological						
Blood triglycerides increased	2 (28.6)	0	0	0	0	0
White blood cell count decreased	0	0	1 (16.7)	0	0	1 (4.8)
Bilirubin conjugated increased	0	0	2 (33.3)	0	2 (33.3)	4 (19.0)
Occult blood positive	0	0	0	3 (50.0)	1 (16.7)	4 (19.0)
Blood bilirubin increased	0	0	1 (16.7)	0	2 (33.3)	3 (14.3)
Blood alkaline phosphatase increased	0	0	0	0	1 (16.7)	1 (4.8)
Neutrophil count decreased	0	0	2 (33.3)	1 (16.7)	0	3 (14.3)
Clinical						
Pain in jaw	0	0	0	0	1 (16.7)	1 (4.8)
Oropharyngeal pain	0	0	0	0	1 (16.7)	1 (4.8)
Abdominal discomfort	0	0	0	0	1 (16.7)	1 (4.8)
Diarrhea	0	0	0	1 (16.7)	0	1 (4.8)
Ventricular extrasystoles	0	1 (33.3)	0	0	0	1 (4.8)
Productive cough	0	0	1 (16.7)	0	0	1 (4.8)
Musculoskeletal discomfort	1 (14.3)	0	0	0	0	0
Treatment-related adverse events, n(%)	2 (28.6)	0	2 (33.3)	4 (66.7)	5 (83.3)	11 (52.4)
Biological						
Blood triglycerides increased	2 (28.6)	0	0	0	0	0
White blood cell count decreased	0	0	1 (16.7)	0	0	1 (4.8)
Bilirubin conjugated increased	0	0	2 (33.3)	0	2 (33.3)	4 (19.0)
Occult blood positive	0	0	0	3 (50.0)	1 (16.7)	4 (19.0)
Blood bilirubin increased	0	0	1 (16.7)	0	2 (33.3)	3 (14.3)
Blood alkaline phosphatase increased	0	0	0	0	1 (16.7)	1 (4.8)
Neutrophil count decreased	0	0	2 (33.3)	1 (16.7)	0	3 (14.3)

Data are n (%). “Treatment-related” is defined as the relationship of an adverse event to the study drug being certain, probable, or possible.

The incidence of treatment-related AEs (TRAEs) was 52.4% (n = 11) and 28.6% (n = 2) in subjects receiving SHR2285 and placebo, respectively (Table 2). TRAEs in the SHR2285 group included increased bilirubin conjugated (19.0%, n = 4), occult blood positive (19.0%, n = 4), decreased neutrophil count (14.3%, n = 3), increased blood bilirubin (14.3%, n = 3), increased blood alkaline phosphatase (4.8%, n = 1), and decreased white blood cell count (4.8%, n = 1).

### PK

All 21 healthy subjects receiving SHR2285 were included in the PK analysis. The PK parameters of both SHR2285 and its main metabolite SHR164471 were summarized descriptively (Table 3, Supplementary Table S1).

**TABLE 3:** Pharmacokinetic parameters of SHR2285.

		SHR2285			
		50 mg (n = 3)	100 mg (n = 6)	200 mg (n = 6)	400 mg (n = 6)
T_max_, h	Median (range)	4.0 (2.0–4.0)	4.0 (2.0–5.0)	3.0 (1.5–4.0)	3.0 (1.5–5.0)
C_max_, ng/mL	Mean ± SD (%CV)	306 ± 129 (42.1)	517 ± 164 (31.8)	789 ± 311 (39.5)	883 ± 105 (11.9)
	GeoMean (%CV)	287 (47.8)	490 (39.8)	747 (35.7)	877 (11.7)
AUC_0-last_, h*ng/mL	Mean ± SD (%CV)	2,450 ± 1,140 (46.5)	5,080 ± 2,570 (50.6)	7,210 ± 2,290 (31.7)	8,490 ± 1,230 (14.5)
	GeoMean (%CV)	2,260 (52.3)	4,540 (56.5)	6,940 (30.6)	8,420 (14.7)
AUC_0-inf,_ h*ng/mL	Mean ± SD (%CV)	2,680 ± 1,220 (45.5)	5,560 ± 2,690 (48.5)	7,480 ± 2,660 (35.5)	9,390 ± 1,240 (13.3)
	GeoMean (%CV)	2,490 (49.8)	5,020 (53.8)	7,140 (34.1)	9,320 (12.7)
t_1/2_, h	Mean ± SD	7.6 ± 2.0	13.2 ± 4.9	12.6 ± 3.4	15.8 ± 7.1
CL/F, L/h	Mean ± SD (%CV)	21.6 ± 9.9 (45.9)	22.1 ± 10.8 (48.8)	29.2 ± 8.6 (29.5)	43.2 ± 5.1 (11.9)
	GeoMean (%CV)	20.1 (49.6)	19.9 (53.8)	28.0 (33.8)	42.9 (12.5)
V_z_/F, L	Mean ± SD (%CV)	251 ± 179 (71.1)	391 ± 164 (41.8)	528 ± 174 (33.0)	996 ± 501 (50.3)
	GeoMean (%CV)	214 (76.2)	360 (49.3)	494 (47.7)	870 (68.1)
MRT_inf_, h	Median (range)	10.3 (8.7–10.8)	16.8 (9.0–21.8)	13.1 (11.7–20.6)	18.2 (8.1–25.1)

AUC_0-inf_, area under the concentration–time curve from zero to time infinity; AUC_0-last_, area under the concentration–time curve from zero to last time of quantifiable concentration; CL/F, apparent clearance; C_max_, maximum plasma concentration; CV, coefficient of variation; GeoMean, geometric mean; MRT, mean residence time to infinity; t_1/2_, terminal elimination half-life; T_max_, time to reach maximum plasma concentration; V_z_/F, apparent volume of distribution.

**FIGURE 2:**
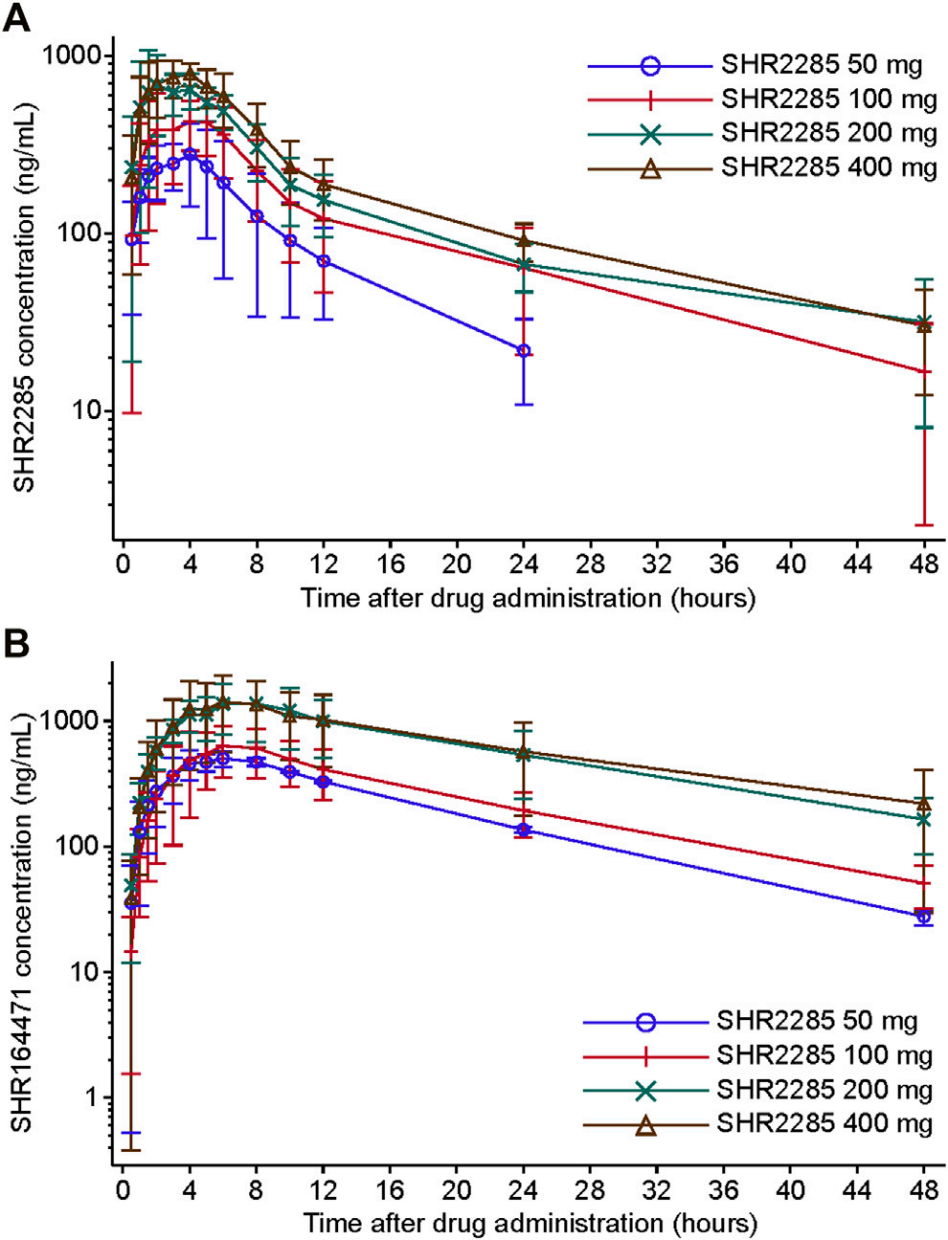
Plasma concentration–time profile of SHR2285 **(A)** and SHR164471 **(B)**. Data are presented as mean (±SD).

SHR2285 was absorbed rapidly after a single oral administration (50, 100, 200, and 400 mg), and the median plasma concentrations in each dose cohort peaked at 3.0–4.0 h postdose (T_max_; Figure 2A, Table 3). The mean T_1/2_ values were similar in the 100-, 200-, and 400-mg groups (13.2 h, 12.6 h, and 15.8 h), while that in the 50-mg group was relatively lower (7.6 h). The plasma exposure of SHR2285 (C_max_, AUC_0-last_, and AUC_0-inf_) increased with higher doses of SHR2285. The mean CL/F and Vz/F values ranged from 21.6–43.2 L/h and 251–996 L, respectively.

The T_max_ of metabolite SHR164471 was slightly longer than that for the parent SHR2285, reaching a peak at 6.0–7.0 h postdose; and the time-varying pattern of SHR164471 concentrations among different dose groups after administration were similar to those for SHR2285 (Supplementary Table S1). The mean t_1/2_ values of SHR164471 were 10.1–14.7 h. SHR164471 exposure (C_max_, AUC_0-last_, and AUC_0-inf_) increased with escalating doses of SHR2285 within the range of 50–200 mg and were similar between the 200- and 400-mg groups (Figure 2B, Supplementary Table S1). The mean CL/F and Vz/F values of SHR164471 in each group ranged from 7.2–35.1 L/h and 105–594 L, respectively.

The sums of AUC_0-inf_ of SHR2285 and SHR164471 in plasma were used to evaluate overall absorption in healthy subjects after a single dose of SHR2285 (Supplementary Table S2). The geometric mean sums of AUC_0-inf_ of SHR2285 and SHR164471 ranged from 17,600 to 67,000 h*nmol/L and increased with higher doses of SHR2285 within the 50- to 200-mg groups. In the 200- and 400 mg groups, the mean sums of AUC_0-inf_ of SHR2285 and SHR164471 were similar, indicating that the absorption ratio of SHR2285 decreased with the increase of the dose, and the sum pharmacological activity of SHR2285 and SHR164471 showed a saturation trend between the 200- and 400 mg doses. Therefore, the dose escalations to 600, 800, and 1,000 mg were terminated.

ANOVAs were conducted to assess the correlations between the several PK parameters of SHR2285 and its metabolites after a single administration (Supplementary Table S3). The C_max_, AUC_last_, and AUC_inf_ of SHR2285 after dose normalization showed intergroup differences. For the metabolite SHR164471, the C_max_ after dose normalization had intergroup differences, whereas AUC_last_ and AUC_inf_ after dose normalization did not. The box–whisker plots revealed positive correlations of C_max_, AUC_last_, and AUC_inf_ with dose (Supplementary Figure S1). These results showed that exposure did not increase linearly with the dose, so no further power model analyses were conducted.

### PD

**FIGURE 3:**
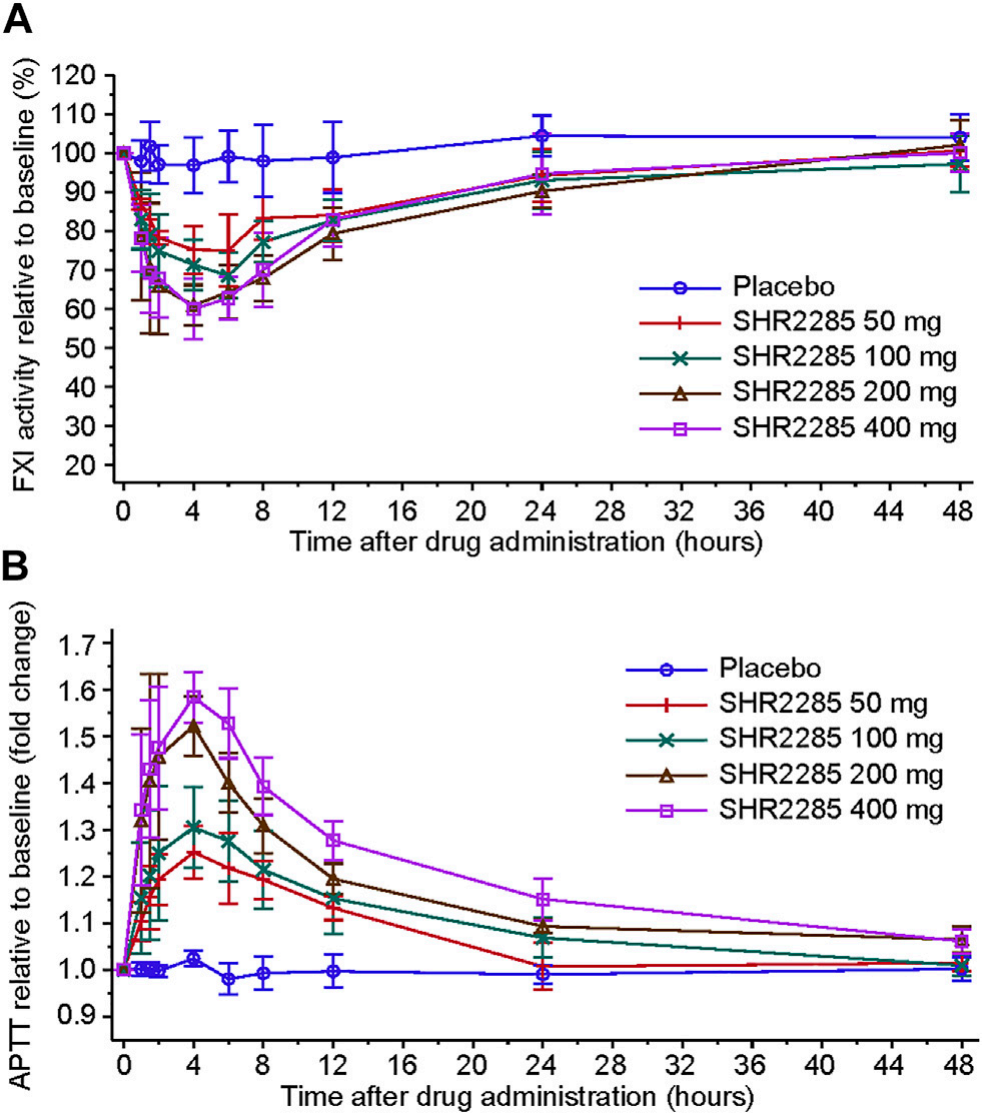
Effects of increasing doses of SHR2285 on FXI **(A)** and APTT **(B)** in healthy volunteers.

PD parameters were analyzed in all the 28 subjects who received either SHR2285 or placebo. After a single oral administration of 50 mg, 100 mg, 200 mg, and 400 mg SHR2285, the serum FXI activity level gradually decreased, and the percentage of decline relative to baseline was higher than that of the placebo group (Figure 3A, Supplementary Table S4). With the elimination of SHR2285, the serum FXI activity level gradually recovered, and the serum FXI activity in each group generally recovered to baseline 48 h after administration. The mean maximum decline of the serum FXI activity level from baseline in 50 mg, 100 mg, 200 mg, and 400 mg group occurred at 6.0 h, 6.0 h, 4.0 h, and 4.0 h postdose, and the mean maximum decline percentage from baseline corresponding to each time point mentioned above was 24.92%, 31.45%, 39.08%, and 40.00%, respectively.

After treatment, the prolongation of APTT to baseline in serum of SHR2285 groups increased simultaneously with the decrease in FXI activity and was higher than that in the placebo group (Figure 3B, Supplementary Table S5). Subsequently, APTT values gradually returned to baseline. The APTT of 50 mg and 100 mg dose groups recovered to the baseline level 24 h after administration, and the APTT of 200- and 400-mg dose groups recovered to the baseline level 48 h after administration. The maximum mean prolongation of APTT relative to baseline in all dose groups appeared at 4.0 h, and the mean maximum prolongation folds relative to baseline corresponding to each time point above were 1.25, 1.31, 1.52, and 1.58, respectively.

The linear regression model demonstrated a certain linear correlation between the decrease of FXI activity from baseline and prolongation of APTT, and the correlation coefficient *R* ^2^ was 0.807 (Supplementary Figure S2). The serum PT and INR levels were not prolonged after SHR2285 administration and were similar to that in the placebo group (Supplementary Figure S3A, B).

### PK and PD Correlations

The *in vitro* PD study showed that both SHR2285 and its main metabolite SHR164471 inhibited human FXI activity and prolonged the APTT. Corrected by the *in vitro* potencies of SHR2285 and SHR164471 on human FXI activity and human blood APTT, the sum of SHR2285/SHR164471 unbound plasma concentration was explored for correlations with FXI activity and APTT. Scatterplots showed that the sum of SHR2285/SHR164471 unbound plasma concentration positively correlated with the percentage decrease in FXI activity and APTT prolongation fold changes from baseline (Supplementary Figure S4A, B).

## Discussion

This was the first clinical evaluation of the small-molecule reversible factor XIa inhibitor SHR2285, which showed inhibition of thrombus formation and no bleeding events in preclinical studies. In this first-in-human study, a single oral dose of SHR2285 was well tolerated and safe in the range of 50–400 mg in healthy subjects, and no bleeding events were observed. The PK results indicated a rapid onset of SHR2285 action, and the PD results demonstrated that SHR2285 could simultaneously prolong APTT and decrease FXI activity.

The present study indicated good tolerability of SHR2285 in healthy subjects after a single dose. No dose-limiting toxicity was observed, and the maximum tolerated dose of SHR2285 was not reached in the range of 50–400 mg. All reported AEs were grade 1 and resolved without special intervention. The incidence of AEs did not increase with higher drug doses. AE and TRAE frequencies were generally similar between the placebo group and the SHR2285 group. Occult blood positivity was noted in four subjects, including three subjects in the 200-mg group and one in the 400-mg group. No abnormalities were observed for routine blood examinations or coagulation functions in those four subjects. All four occult blood positive events were sporadic, with mild symptoms, no other bleeding symptoms and regressed without intervention Notably, there was no sign of increased bleeding events in this study, as compared with the traditional anticoagulant factor II inhibitor warfarin which carries an obvious bleeding risk, there might be less need to monitor the clinical indicators associated with bleeding after SHR2285 administration. Compared with low-molecular-weight heparin (e.g., injectable enoxaparin), SHR2285 has the advantage of convenient oral administration. Collectively, the SHR2285 safety data suggest that it might be a useful new anticoagulant treatment.

In patients with atrial fibrillation who underwent percutaneous coronary intervention (PCI), a meta-analysis showed that the addition of anticoagulant drugs (such as warfarin) to dual antiplatelet therapy was associated with a high risk of bleeding (Gragnano et al., 2019). In our first-in-human study, no bleeding events of SHR2285 were reported, indicating that SHR2285 might be a potential choice of anticoagulants for patients with atrial fibrillation who underwent PCI and required a combination of dual antiplatelet therapy with the anticoagulant drug.

The PK profile of SHR2285 was comparable with other small molecular FXIa inhibitors such as ONO-7684, BMS-962212, and BAY 243334 (Perera et al., 2018; Beale et al., 2021; Thomas et al., 2021). The rapid plasma distribution of SHR2285 supports further investigations of this drug for acute anticoagulant therapy because drugs with a rapid increase in exposure may be more suitable for such subjects. The mean T_1/2_ of the 50-mg dose group (7.6 h) was lower than that of the other three dose groups. This phenomenon was because the blood concentration of 50 mg at 48 h was below the limit of quantification (5 ng/ml), the entire elimination phase was not captured, and the small sample size of the 50-mg group.

The PD parameters of SHR2285 in this clinical study were also similar to those for previously reported oral small-molecule FXIa inhibitors (Perera et al., 2018; Beale et al., 2021; Thomas et al., 2021). In this study, SHR2285 administration prolonged APTT, which was similar to the results of preclinical animal model studies. In addition, FXI activity inhibition by SHR2285 was synchronized with prolonged APTT. APTT can be used to monitor heparin levels in patients requiring anticoagulation therapy, and it is also used as a biomarker to reflect the anticoagulant activities of factor XIa inhibitors (Smythe et al., 2001; Ignjatovic, 2013). The remission of thromboembolic events after SHR2285 administration needs to be explored in further clinical studies. With decreased FXI activity and prolonged APTT, PT and INR were not affected following SHR2285 administration. This finding was in line with the safety results showing no bleeding events. Currently, it is not clear whether a larger decrease in FXI levels provides more effective protection against thromboembolism. Some studies showed that severe FXI deficiency with residual FXI: C (FXI coagulant activity) < 15% could prevent stroke and venous thromboembolism (Salomon et al., 2008; Salomon et al., 2011), while others reported that even moderate FXI deficiency (residual FXI activity of 30%–50%) could prevent venous thrombosis and cardiovascular events (Preis et al., 2017).

We found that the sum of SHR2285/SHR164471 unbound plasma concentration corrected by the *in vitro* potency was positively associated with decreased FXI activity and prolonged APTT. Since the PK/PD relationship is a key link between optimal drug dosing and clinical outcomes, characterizing the PK/PD correlation could be helpful to guide the selection of doses and schedules in further clinical development.

The major limitation of this work is that it was a phase one study conducted in healthy subjects, so the anticoagulant efficacy is unknown. However, our safety, PK, and PD results support further investigation of SHR2285 in patients who require anticoagulation. In addition, although the sample size of this study meets the requirement of a first-in-human trial, the relatively small number of subjects may introduce certain bias in interpreting the results.

In conclusion, SHR2285 was well tolerated and safe when administrated as a single oral dose in healthy subjects. The PK results revealed its rapid onset of action. The synchronized decrease in FXI activity and increase in APTT were observed over the studied dose range. Collectively, these results support further clinical development of SHR2285 as an effective oral anticoagulant.

## Data Availability

The datasets presented in this article are not readily available. The datasets used and/or analyzed during the current study are available from the corresponding author on reasonable request after the product and indication has been approved by major health authorities. Data may be requested 24 months after study completion. Qualified researchers should submit a proposal to the corresponding author outlining the reasons for requiring the data. The leading clinical site and sponsor will check whether the request is subject to any intellectual property restriction. The use of data must also comply with the requirements of Human Genetics Resources Administration of China and other country or region-specific regulations. A signed data access agreement with the sponsor is required before accessing shared data. Requests to access the datasets should be directed to shuyangzhang103@nrdrs.org.
