# Supplementary material for: First-In-Human Study to Assess the Safety, Pharmacokinetics, and Pharmacodynamics of SHR2285, a Small-Molecule Factor XIa Inhibitor in Healthy Subjects
Source: Front Pharmacol. 2022 Feb 10;13:821363. doi: 10.3389/fphar.2022.821363 (PMC8866703; doi:10.3389/fphar.2022.821363)
Supplement: Supplementary file 1 [file DataSheet1.docx]

**Supplement**

Supplement to: Rui Chen, et al. First-in-human study to assess the safety, pharmacokinetics, and pharmacodynamics of SHR2285, a small-molecule factor XIa inhibitor in healthy subjects

**Table of contents**

[Figure S1. Box-whisker plots of selected pharmacokinetic parameters in different dose cohorts. 2](#_Toc92965773)

[Figure S2. Correlation between decreased FXI activity from baseline and APTT prolongation 3](#_Toc92965774)

[Figure S3. Percentages of PT (A) and INR (B) changes from baseline. 4](#_Toc92965775)

[Figure S4. Correlation of SHR2285/SHR164471 unbound plasma concentrations with decrease of FXI activity (A) and APTT prolongation (B). 5](#_Toc92965776)

[Table S1. PK parameters of SHR164471. 6](#_Toc92965777)

[Table S2. The AUC_0-inf_ sums of SHR2285 and SHR164471. 8](#_Toc92965778)

[Table S3. ANOVA on the logarithm of selected PK parameters of SHR2285 and SHR164471 in plasma. 9](#_Toc92965779)

[Table S4. Percentages of changes of FXI activity from baseline. 10](#_Toc92965780)

[Table S5. Percentages of changes of APTT activity from baseline. 11](#_Toc92965781)

## Figure S1. Box-whisker plots of selected pharmacokinetic parameters in different dose cohorts.

(A) C_max_ of SHR2285; (B) AUC_0-last_ of SHR2285; (C) AUC_0-inf_ of SHR2285; (D) C_max_ of SHR164471; (E) AUC_0-last_ of SHR164471; (F) AUC_0-inf_ of SHR164471.


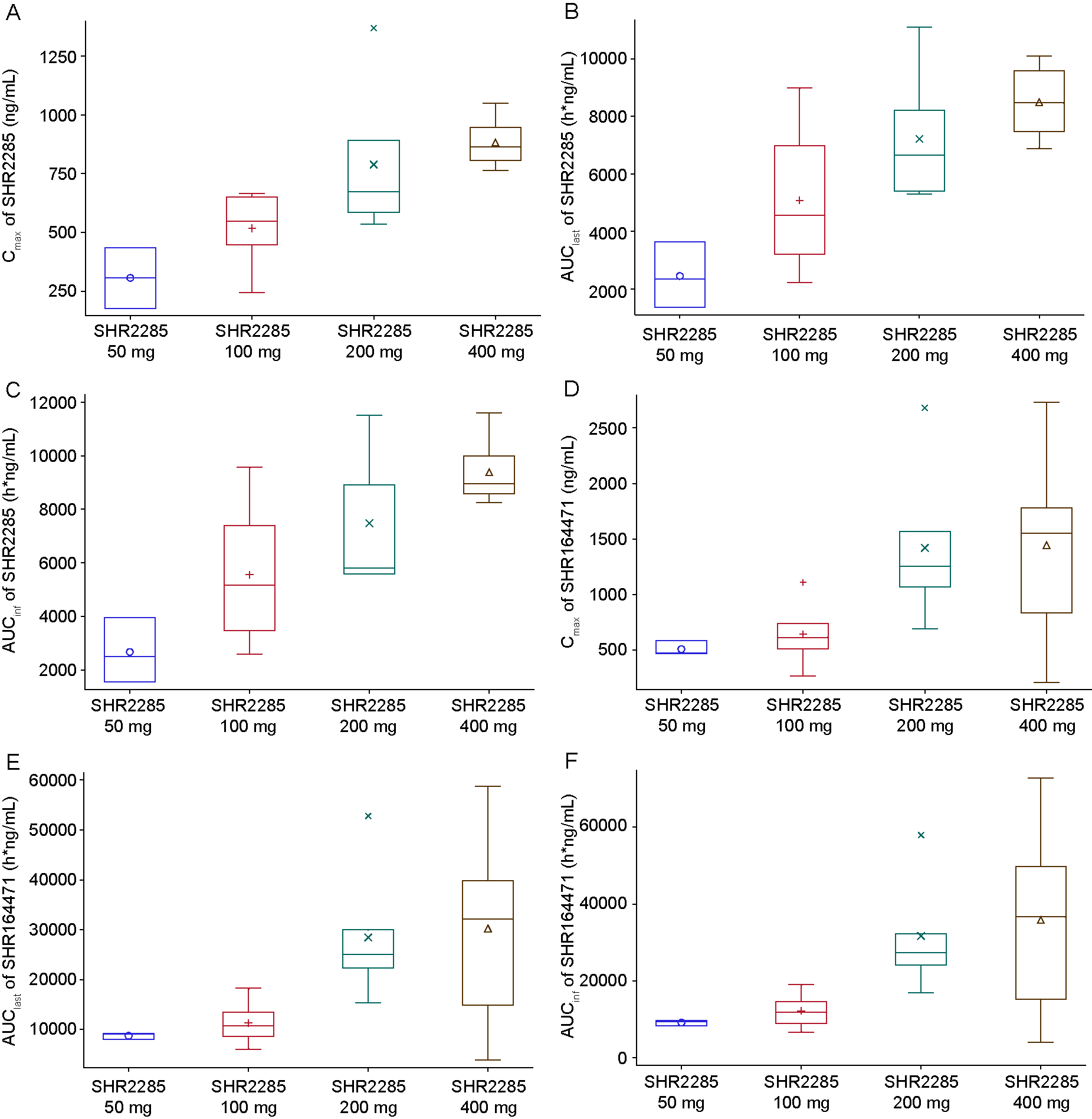


## Figure S2. Correlation between decreased FXI activity from baseline and APTT prolongation


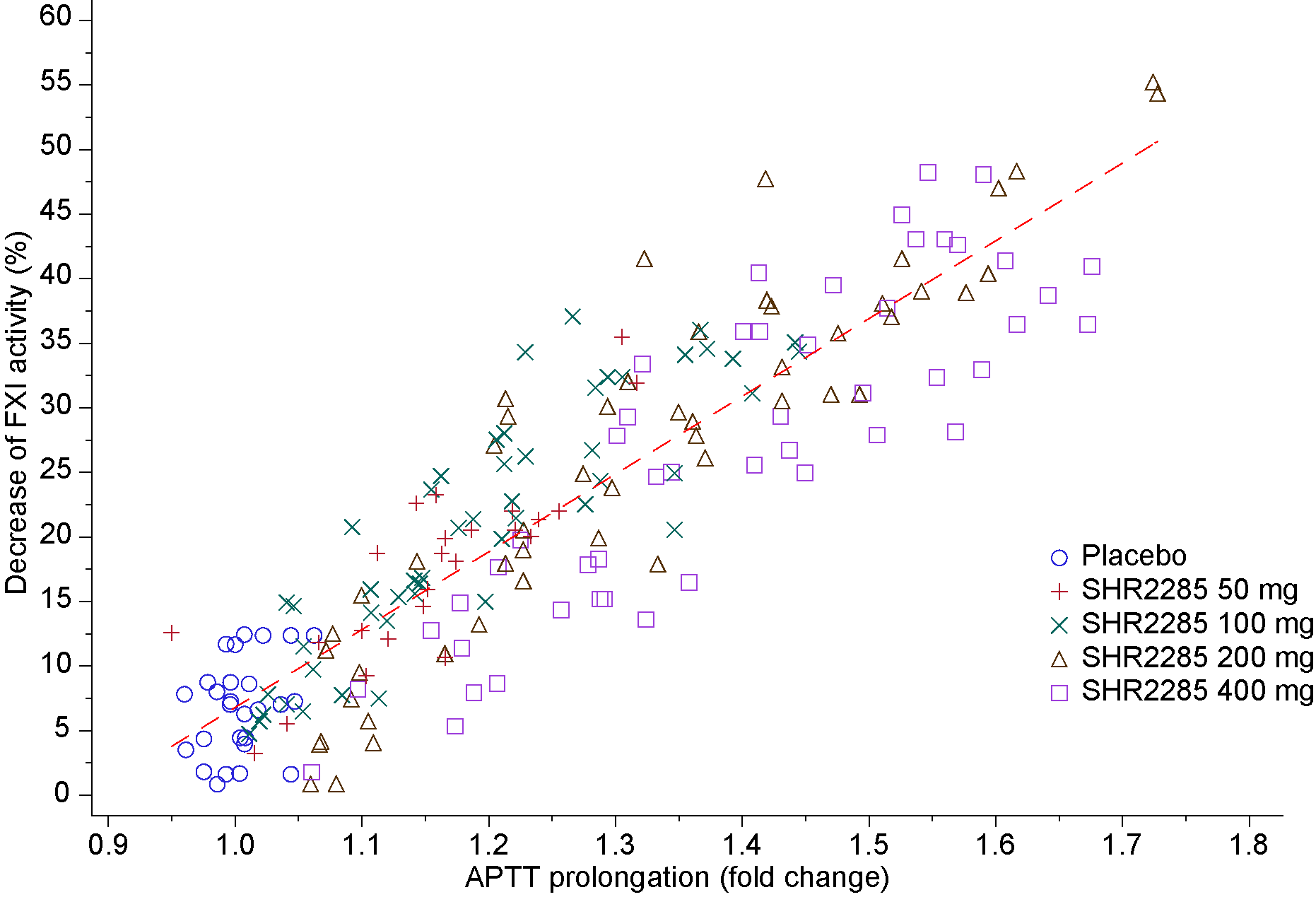


## Figure S3. Percentages of PT (A) and INR (B) changes from baseline.


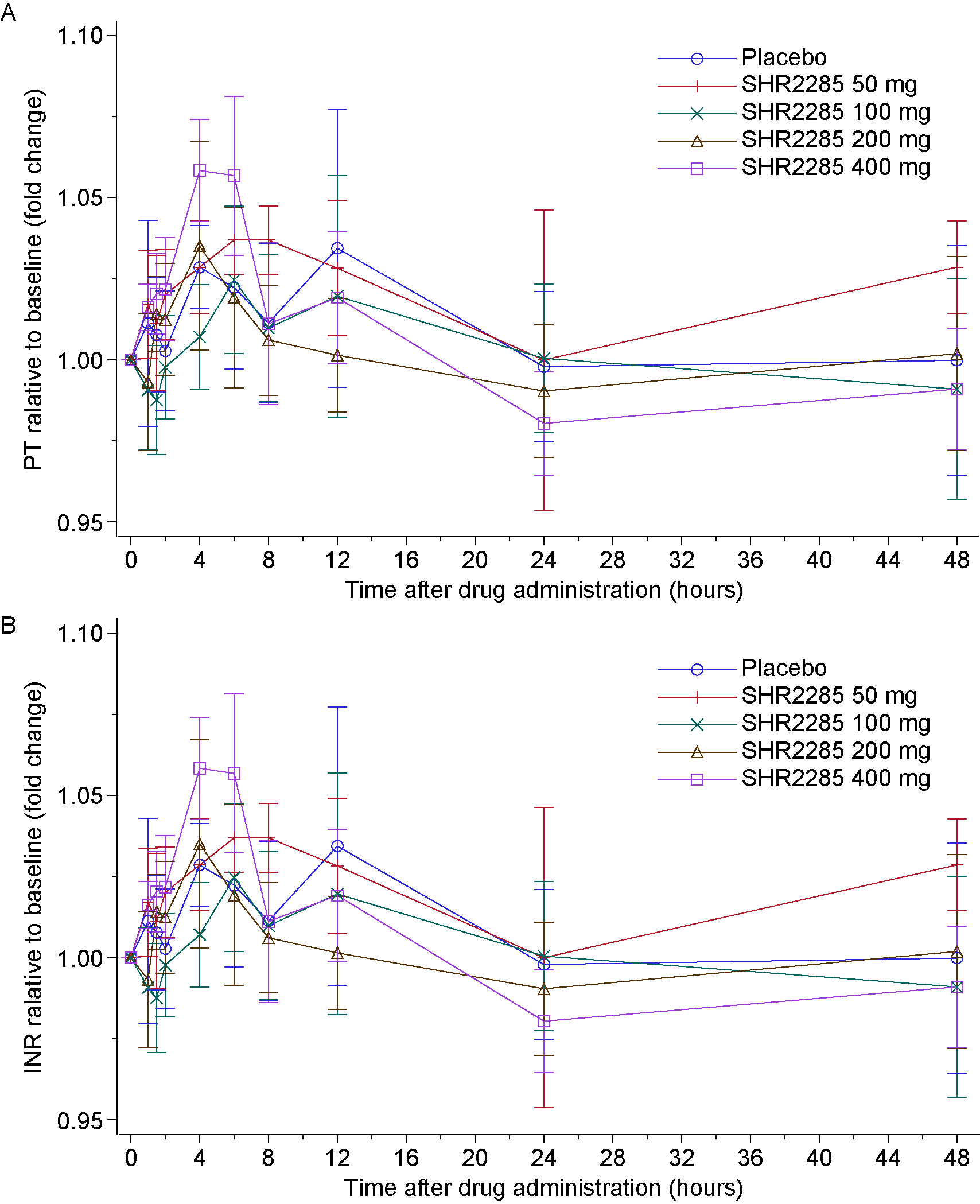


## Figure S4. Correlation of SHR2285/SHR164471 unbound plasma concentrations with decrease of FXI activity (A) and APTT prolongation (B).

In panel A, the x-axis indicates sum of unbounded plasma concentration of SHR2285 and SHR164471*IC_50_ of SHR2285 on FXI activity/IC_50_ of SHR164471 on FXI activity. In panel B, the x-axis represents the sum of unbounded plasma concentration of SHR2285 and SHR164471*CT_2.0_ of SHR2285 on APTT/CT_2.0_ of SHR164471 on APTT. The plasma-protein binding rate was measured using rapid equilibrium dialysis. Unbound plasma concentration=total plasma concentration*(1–protein binding%).


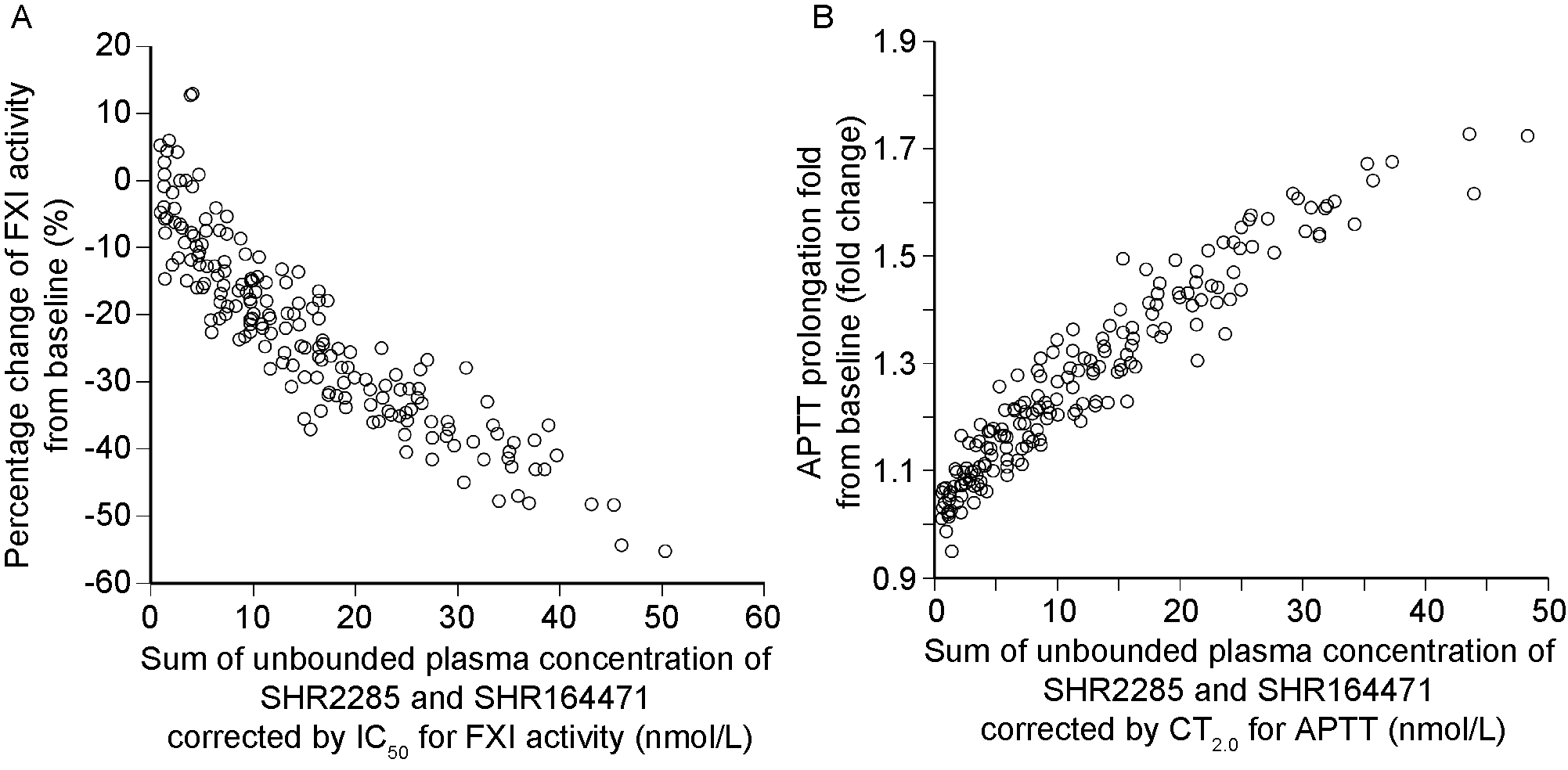


## Table S1. PK parameters of SHR164471.

|  | | **50 mg (n=3)** | **100 mg (n=6)** | **200 mg (n=6)** | **400 mg (n=6)** |
| --- | --- | --- | --- | --- | --- |
| T_max_, h | Median (range) | 6.0 (6.0-8.0) | 6.9 (4.0-8.0) | 7.0 (6.0-8.0) | 6.0 (6.0-8.0) |
| C_max_, ng/mL | Mean±SD (%CV) | 511±65.0 (12.7) | 645±281 (43.5) | 1420±681 (48.0) | 1450±859 (59.4) |
|  | GeoMean (%CV) | 508 (12.4) | 592 (49.7) | 1300 (47.0) | 1130 (112.3) |
| AUC_0-last_, h*ng/mL | Mean±SD (%CV) | 8760±664 (7.6) | 11300±4370 (38.6) | 28500±12900 (45.2) | 30300±19300 (63.7) |
|  | GeoMean (%CV) | 8740 (7.8) | 10600 (40.9) | 26400 (42.3) | 22700 (126.6) |
| AUC_0-inf,_ h*ng/mL | Mean±SD (%CV) | 9170±724 (7.9) | 12200±4500 (36.8) | 31700±15700 (49.4) | 35900±24600 (68.5) |
|  | GeoMean (%CV) | 9150 (8.1) | 11500 (39.3) | 29100 (47.6) | 25900 (140.0) |
| t_1/2_, h | Mean±SD | 10.1±0.6 | 12.4±2.3 | 12.6±0.7 | 14.7±4.7 |
| CL/F, L/h | Mean±SD (%CV) | 7.2±0.6 (8.3) | 12.2±4.7 (38.3) | 9.8±4.0 (41.2) | 35.1±47.0 (134.0) |
|  | GeoMean (%CV) | 7.2 (8.1) | 11.5 (39.3) | 9.1 (47.5) | 20.4 (140.1) |
| V_z_/F, L | Mean±SD (%CV) | 105±6.2 (5.9) | 221±103 (46.5) | 179±76.3 (42.6) | 594±702 (118.1) |
|  | GeoMean (%CV) | 105 (5.9) | 202 (50.2) | 165 (49.3) | 413 (96.9) |
| MRT_inf_, h | Median (range) | 16.8 (16.2-17.4) | 21.9 (15.8-22.6) | 21.0 (19.6-22.3) | 23.6 (15.5-31.3) |

AUC_0-inf_, area under the concentration time curve from zero to time infinity; AUC_0-last_, area under the concentration time curve from zero to last time of quantifiable concentration; CL/F, apparent clearance; C_max_, maximum plasma concentration; MRT, mean residence time to infinity; t_1/2_, terminal elimination half-life; T_max_, time to reach maximum plasma concentration; V_z_/F, apparent volume of distribution. The CL/F and V_z_/F of SHR164471 were calculated by converting the dosage of SHR2285 into an equimolar dose of SHR164471.

## Table S2. The AUC_0-inf_ sums of SHR2285 and SHR164471.

|  | | **50 mg (n=3)** | **100 mg (n=6)** | **200 mg (n=6)** | **400 mg (n=6)** |
| --- | --- | --- | --- | --- | --- |
| AUC_0-inf,_ h*nmol/L | Mean±SD (%CV) | 17600±1270 (7.2) | 27200±5990 (22.1) | 57700±19400 (33.6) | 67000±34100 (50.9) |
|  | GeoMean (%CV) | 17600 (7.2) | 26500 (24.5) | 55500 (30.3) | 58600 (67.0) |

AUC_0-inf_, area under the concentration time curve from zero to time infinity. AUC_0-inf_ of SHR2285 and SHR164471 was corrected by respective molecular weight.

## Table S3. ANOVA on the logarithm of selected PK parameters of SHR2285 and SHR164471 in plasma.

| **Parameters** | **Model factor** | **Degrees of freedom** | **Mean sum of squares** | **Mean square** | **F value** |
| --- | --- | --- | --- | --- | --- |
| SHR2285 | | | | | |
| ln(AUC_0-inf_), h*ng/mL/dose | Dose | 3 | 2.1070 | 0.7023 | 5.0332 |
|  | Error | 16 | 2.2326 | 0.1395 | - |
| ln(AUC_last_), h*ng/mL/dose | Dose | 3 | 2.1314 | 0.7105 | 4.9823 |
|  | Error | 17 | 2.4242 | 0.1426 | - |
| ln(C_max_), ng/mL/dose | Dose | 3 | 2.6934 | 0.8978 | 8.4138 |
|  | Error | 17 | 1.8140 | 0.1067 | - |
| SHR164471 | | | | | |
| ln(AUC_0-inf_), h*ng/mL/dose | Dose | 3 | 2.8647 | 0.9549 | 2.1906 |
|  | Error | 16 | 6.9745 | 0.4359 | - |
| ln(AUC_last_), h*ng/mL/dose | Dose | 3 | 3.3391 | 1.1130 | 2.9595 |
|  | Error | 17 | 6.3934 | 0.3761 | - |
| ln(C_max_), ng/mL/dose | Dose | 3 | 3.9757 | 1.3252 | 3.6256 |
|  | Error | 17 | 6.2139 | 0.3655 | - |

ANOVA, analysis of variance; AUC_0-inf_, area under the concentration time curve from zero to time infinity; AUC_last_, area under the concentration time curve from zero to last time of quantifiable concentration; C_max_, maximum plasma concentration.

## Table S4. Percentages of changes of FXI activity from baseline.

|  | **Placebo (n=7)** | **50 mg (n=3)** | **100 mg (n=6)** | **200 mg (n=6)** | **400 mg (n=6)** |
| --- | --- | --- | --- | --- | --- |
| 1 h | 5.67±3.78 | 13.07±1.42 | 17.03±7.59 | 21.33±16.38 | 21.77±8.67 |
| 1.5 h | 3.74±4.35 | 16.96±4.26 | 20.88±10.48 | 29.55±16.69 | 30.72±10.19 |
| 2 h | 5.47±2.50 | 21.71±1.69 | 25.11±9.32 | 34.20±12.28 | 32.06±10.24 |
| 4 h | 8.32±2.72 | 24.82±6.20 | 28.68±6.55 | 39.08±5.22 | 40.00±7.65 |
| 6 h | 9.46±3.11 | 24.92±9.21 | 31.45±5.86 | 35.68±6.86 | 37.25±5.51 |
| 8 h | 8.85±4.09 | 16.71±5.48 | 22.77±5.31 | 32.15±5.93 | 29.97±9.43 |
| 12 h | 9.75±4.60 | 15.93±6.69 | 17.28±5.28 | 20.67±6.71 | 17.01±6.96 |
| 24 h | 3.96±0 | 9.04±4.98 | 9.58±3.79 | 9.75±4.27 | 11.09±4.09 |
| 48 h | 1.72±0.13 | 3.25±0 | 8.37±5.46 | 2.98±1.84 | 5.00±4.55 |

Data are mean±SD.

## Table S5. Percentages of changes of APTT activity from baseline.

|  | **Placebo (n=7)** | **50 mg (n=3)** | **100 mg (n=6)** | **200 mg (n=6)** | **400 mg (n=6)** |
| --- | --- | --- | --- | --- | --- |
| 1 h | 1.00±0.02 | 1.10±0.04 | 1.15±0.12 | 1.32±0.20 | 1.34±0.16 |
| 1.5 h | 1.00±0.01 | 1.16±0.07 | 1.20±0.14 | 1.40±0.23 | 1.43±0.15 |
| 2 h | 1.00±0.01 | 1.19±0.05 | 1.25±0.14 | 1.46±0.18 | 1.48±0.13 |
| 4 h | 1.02±0.02 | 1.25±0.06 | 1.31±0.09 | 1.52±0.06 | 1.58±0.06 |
| 6 h | 0.98±0.03 | 1.22±0.08 | 1.28±0.09 | 1.40±0.06 | 1.53±0.07 |
| 8 h | 0.99±0.04 | 1.19±0.04 | 1.21±0.08 | 1.31±0.06 | 1.39±0.06 |
| 12 h | 1.00±0.04 | 1.13±0.03 | 1.15±0.08 | 1.19±0.03 | 1.28±0.04 |
| 24 h | 0.99±0.02 | 1.01±0.05 | 1.07±0.04 | 1.09±0.02 | 1.15±0.05 |
| 48 h | 1.00±0.03 | 1.02±0.02 | 1.01±0.02 | 1.06±0.03 | 1.06±0.03 |

Data are mean±SD.
